# Bullous Pemphigoid in Patients Receiving Immune-Checkpoint Inhibitors and Psoriatic Patients—Focus on Clinical and Histopathological Variation

**DOI:** 10.3390/dermatopathology9010010

**Published:** 2022-03-18

**Authors:** Dennis Niebel, Dagmar Wilsmann-Theis, Thomas Bieber, Mark Berneburg, Joerg Wenzel, Christine Braegelmann

**Affiliations:** 1 Department of Dermatology, University Hospital Regensburg, 93053 Regensburg, Germany; mark.berneburg@ukr.de; 2 Department of Dermatology and Allergy, University Hospital Bonn, 53105 Bonn, Germany; dagmar.wilsmann-theis@ukbonn.de (D.W.-T.); thomas.bieber@ukbonn.de (T.B.); joerg.wenzel@ukbonn.de (J.W.); christine.braegelmann@ukbonn.de (C.B.)

**Keywords:** pemphigoid, bullous, skin diseases, vesiculobullous, psoriasis, programmed cell death 1 receptor, immune checkpoint inhibitors

## Abstract

Background: The most common autoimmune blistering disease, bullous pemphigoid (BP), shows an increased prevalence in psoriatic patients and oncologic patients undergoing immune-checkpoint blockade (ICB). Even though the same autoantigens (BP180/BP230) are detectable, it remains obscure whether clinical or histopathological differences exist between these different groups of BP patients. In this study, we strived to analyze this matter based on own data and previously published reports. Methods: We performed an institutional chart review from 2010–2020 to identify BP patients with psoriasis (*n* = 6) or underlying ICB (*n* = 4) and matched them with idiopathic cases of BP (*n* = 33). We compared clinical characteristics, subtypes, and dermatopathological determinants (e.g., tissue eosinophilia/neutrophilia, papillary edema, lymphocytic infiltration) among the groups. Results: ICB-associated BP affects men more often and might show mucosal involvement more frequently. We found no statistically significant dermatopathological differences among the groups. Conclusions: Clinicians should be aware of an increased risk of BP in patients with psoriasis and oncologic patients receiving ICB; atypical pruritic skin lesions should prompt a workup including a skin biopsy for histopathology and direct immunofluorescence in these patients. Larger studies might be necessary to detect slight dermatopathological variation.

## 1. Introduction

Among the dermatopathological conditions defined by a vesiculo-bullous reaction pattern, autoimmune blistering diseases (AIBS) are common. Based on the level of splitting, pemphigus group diseases showing intraepidermal clefting are distinguished from pemphigoid group diseases, which display subepidermal clefting [1]. Circulating autoantibodies are causative in both disease groups and detectable via different immunological techniques, i.e., direct or indirect immunofluorescence (DIF/IF), enzyme-linked sorbent assay (ELISA), or immunoblotting. These autoantibodies target desmogleins as a component of desmosomes in pemphigus and bullous pemphigoid antigen 2 (BP180) or bullous pemphigoid antigen 1 (BP230) as elements of hemidesmosomes in bullous pemphigoid (BP).

While fully developed lesions in BP are clinically defined by tense bullae on a reddish base in flexural sides of extremities and the trunk, early signs of disease may appear as urticarial or eczematous rash (prodromal BP) [2]. Intense pruritus is a hallmark symptom. Like most autoimmune diseases, BP shows a tendency to affect female patients more often than male patients [3]. Other variants are prurigo-type BP (resulting from prolonged rubbing in accessible body regions), localized BP type (most often affecting the lower extremities), vesicular type (clinically resembling dermatitis herpetiformis), vegetating type (primarily affecting the groins), and polymorphic type (displaying features of various AIBS) [1].

Histologically, BP is commonly defined by marked papillary edema and an inflammatory infiltrate of eosinophils and neutrophils, with the latter being more scant. Variable numbers of lymphocytes and histiocytes are intermingled around the superficial vascular plexus. Depending on the number of eosinophils, flame figures may be present as a sign of degradation of collagen fibers. The presence of a subepidermal cleft pushing the basal membrane to the bottom of the blister with an accompanying inflammatory infiltrate are decisively indicative of BP. However, as all aforementioned criteria are variable, and as lesions without underlying edema may appear cell-poor in conventional microscopy, it may be difficult to distinguish between different subepidermal AIBS (e.g., epidermolysis bullosa aquisita) [1]. Therefore, diagnosis should be routinely supported by IF and DIF. Typically, linear deposits of C3 and immunoglobulins (most often IgG) along the dermo-epidermal junction are detected [4]. The appearance of BP with the above-mentioned consistent immunofluorescence features in fully developed lesions of lichen planus has been termed lichen planus pemphigoides. This disease entity should be separated from bullous lichen planus, which is negative in IF/DIF; vesicles or bullae result from massive vacuolar alteration in this instance [2].

Association studies found an increased risk for BP with malignancy [5], diabetes mellitus [6], and intake of various drugs (e.g., dipeptidyl 4 inhibitors) [7]. Over the last years, reports about BP induced by immune-checkpoint blockade (ICB) [8] mounted, which will be discussed further on.

### 1.1. Psoriasis

Psoriasis comprises a spectrum of chronic inflammatory skin conditions and affects up to 1% of the general population [2]. It is considered a systemic inflammatory disease featuring numerous comorbidities [9]. The incidence of BP is increased in psoriatic patients, which has been repeatedly reported based on large cohort studies [10,11,12]. Some authors in the past regarded this phenomenon an incidental finding or as a chance occurrence. However, it is now well-established that both psoriatic inflammation itself and certain treatment modalities including UV therapy [13] may elicit eruptions of BP [14,15]. Hypothetically, increased epithelial turnover in line with chronic inflammation and degradation of components of the basal membrane might pave the way to antigen presentation and consecutive autoantibody production. Another explanation is that psoriatic inflammation is associated with senescence of the extracellular matrix and shortened telomere lengths, which carries a risk of BP induction [16]. Although in most cases psoriasis precedes BP, there are few cases in which BP precedes psoriasis [17]. It remains dubious whether psoriasis induces BP, and if so, how a switch from a Th1/Th17- to a Th2-dominated inflammatory milieu is established [18]. Interestingly, there is a male predominance in psoriatic patients who develop BP, and patients with pustular variants seem to be at a higher risk for AIBS [19]; the exact etiological mechanisms remain to be discovered [16].

Generally, BP in psoriatic patients and BP in non-psoriatic patients are considered clinically equivalent. Yet, based on numerous pathophysiological abnormalities in psoriatic skin, histopathological differences seem plausible. Abundance of IL17A in psoriatic skin might stimulate pronounced neutrophilic recruitment. This leads to the hypothesis that BP in psoriatic patients may be accompanied by a more neutrophilic infiltrate when compared to idiopathic BP, as recently investigated by a Japanese group [20].

### 1.2. Immune Checkpoint Blockade (ICB) and Autoimmune Blistering Skin Diseases (AIBS)

Co-inhibitory monoclonal antibodies of immune checkpoints on antigen presenting cells and T cells, i.e., immune checkpoint inhibitors (ICI), have revolutionized treatment of different types of cancer. Inhibitors of programmed cell death protein 1 (PD1) and programmed cell death protein 1 ligand 1 (PDL1), among which nivolumab, pembrolizumab, cemiplimab, atezolizumab and avelumab are most commonly used, may shift the cellular immune response pattern towards a Th1 phenotype [21]. Not surprisingly, psoriasiform and lichenoid skin reactions are a common side effect of these drugs that occur in a large number of patients to a variable extent [22,23]. Rarely, but repeatedly, AIBS including BP arise in the course of PD1/PDL1 based ICB [24]. Reasons for that might include tumoral expression of BP180 leading to cross-reactivity [25]. Another hypothesis is that PD1 therapy might inhibit regulatory T cells to result in dysregulation of B cells with consecutive autoantibody production [24]. Interestingly, malignancies most commonly treated with ICB, i.e., melanoma, might be associated with BP itself [26]. Risk factors for development of ICB-related cutaneous adverse events in general have been published recently based on real-world data and include diagnosis of melanoma or renal cell carcinoma and patients receiving combinatorial ICB with antibodies directed at cytotoxic T-lymphocyte-associated protein 4 (CTLA4) [27].

Up until this date, differences between idiopathic BP and ICB-associated BP have not been characterized satisfactorily on a clinical, histopathological and functional level [28]. We established the hypothesis that ICB-associated BP could be reflected more accurately by BP of psoriatic patients when compared to idiopathic BP cases based on an underlying ICB-derived Th1 shift. The reason to conduct this study was to compile available data and revise our own patient records with regard to clinical and histopathological features within these patient groups.

## 2. Materials and Methods

### 2.1. Patients and Inclusion Criteria

We performed a single center retrospective analysis regarding all histopathologic specimens of the Department of Dermatology and Allergy of the University Hospital Bonn, including patients from 2010 to 2020. Full text search terms “pemphigoid” or “BP” combined with “psoriasis”, “pso”, “PD1”, “pembrolizumab”, “nivolumab” or “immune checkpoint” were used to identify patients suffering from both conditions in the institutional software (PathoPro Version 9.0.9070, IFMS GmbH, Saarbrücken, Germany). Only patients with DIF/IF confirmed BP and available full blood count at the time of the biopsy were included; the first confirmatory biopsy of the patient was included in cases of multiple biopsies. *n* = 6 patients with BP and underlying psoriatic diathesis and *n* = 4 patients with BP with underlying ICB were identified and included in this study. A control group (*n* = 33) was assembled with the most recent institutional cases of BP without psoriatic diathesis or ICB starting in 2019.

### 2.2. Chart Review

We performed a thorough chart review with regard to multiple clinical characteristics and risk factors for AIBS and specific treatment regarding BP. We excluded further appraisal of therapeutic response in this study, as data was not sufficient to do so.

### 2.3. Histopathology

Sections were processed according to standard protocol and stained with hematoxylin–eosin. We manually assessed tissue eosinophil and neutrophil count in four randomly selected high-power fields from different sections around the dermo-epidermal junction (HPF, ×400). The examination was performed independently by two experienced dermatopathologists (D.N. and J.W.). If their counts were consistent within a range of 20%, the mean number of eosinophils/neutrophils was calculated and used for subsequent analyses. Inconsistent results could be resolved using a discussion microscope. A ratio of mean eosinophil count divided by mean neutrophil count was calculated to determine the predominant cell type (number > 1 signifying predominance of eosinophils, number < 1 signifying predominance of neutrophils). Additionally, we semi-quantitatively analyzed further histopathological determinants of BP, i.e., papillary edema and perivascular lymphocytic infiltrate (0 = absent, 1 = low, 2 = moderate, 3 = strong). Presence or absence of eosinophilic spongiosis, flame figures and epidermal necrosis was also noted (0 = absent, 1 = present).

### 2.4. Statistical Analysis

We performed descriptive statistical analyses with MS Excel 2010 Version 14.0.7268.5000 (Microsoft Corp., Redmond, WA, USA) and Prism (GraphPad Sofware, San Diego, CA, USA). Mean values are given ± standard deviation. The Mann–Whitney test was applied to determine statistical significance; *p*-levels < 0.05 were considered significant.

### 2.5. Microscope and Camera

All photomicrographs were captured using a Leica DM LB microscope with an attached KY-F75U digital camera (JVCKENWOOD Deutschland GmbH, Bad Vilbel, Germany). DISKUS software version 4.60.1171 (Technisches Büro Hilgers, Königswinter, Germany) was used; we refrained from digital enhancement. Figures were created using MS Office Professional Plus 2010 Version 14.0.7268.5000 (Microsoft Corp., Redmond, WA, USA).

### 2.6. Literature Review

To compare our data with previously published case reports featuring BP in patients receiving ICB, we performed a structured literature review in May 2021. We searched PubMed database using the search string: (((“bullous pemphigoid” [All Fields]) AND ((PD1) OR (CTLA4) OR (immune checkpoint) OR (immunotherapy))), which yielded 153 results. Available reviews were manually searched for other cited case reports, which were then also included. We incorporated papers in the English or German language only. Ultimately, we were able to include 55 manuscripts accounting for 71 case reports and analyzed the reported clinical and histopathological features.

## 3. Results

### 3.1. Clinical Characteristics

We identified six BP patients with concomitant psoriatic diathesis with an even gender proportion (Table 1). Five patients had long-standing psoriasis vulgaris and one patient suffered from generalized pustular psoriasis. Out of four patients with ICB-associated BP, 75% were male. Two patients suffered from metastatic melanoma and one patient had metastatic non-squamous cell carcinoma of the lung (NSCLC) who received nivolumab monotherapy. One patient suffered from metastatic renal cell carcinoma (RCC) and received pembrolizumab monotherapy. The mean age was notably lower when compared to the other groups, with a mean of 73 years. Half of the patients suffered from mucosal involvement. All patients described pruritus to a varying degree.

As a control group, we included 33 patients with BP without psoriatic diathesis or preceding ICB. Gender distribution differed from the other two groups, as there was a female predominance of 61%. Only two patients suffered from mucosal involvement, and all but one patient (Mediterranean) were of central European descent.

**Figure 1:**
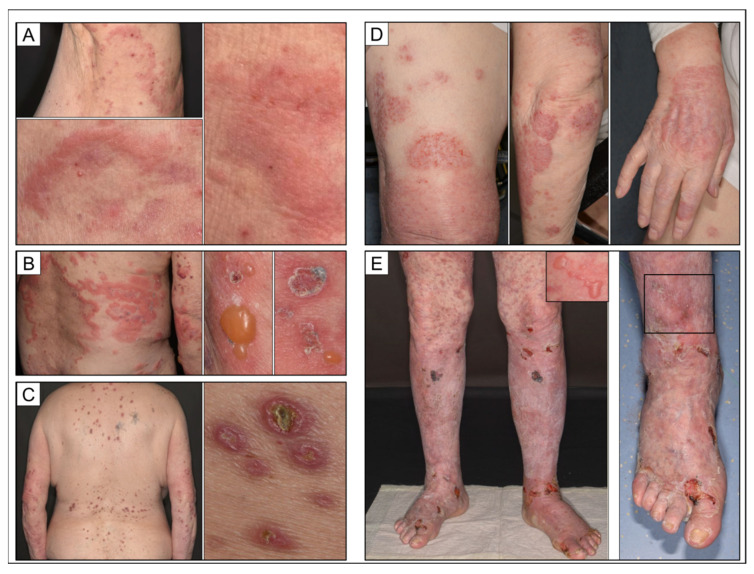
Clinical synopsis of different manifestations of bullous pemphigoid. (**A**) Prodromal stage of bullous pemphigoid featuring urticarial plaques without blistering. (**B**) Fully developed typical bullous pemphigoid featuring more livid erythematous urticarial plaques with tense blisters in the peripheral rim; older lesions exhibit crusts. (**C**) Clinical variant of bullous pemphigoid showing features of chronic prurigo while lacking vesicles; singular lesions display an umbilicated aspect and are restricted to areas amenable to persistent rubbing. (**D**) Bullous pemphigoid in a psoriatic patient; distinct eczematous clinical picture restricted to preexisting psoriatic plaques. (**E**) Acute pustular exacerbation of long-standing papulosquamous psoriasis after psoralen ultraviolet A (PUVA) therapy. Note concomitant tense blisters and pustules in different areas (rectangles).

In order to define differences in the clinical appearance between idiopathic cases of BP and BP in psoriatic patients or patients receiving ICB, we defined categories as follows: typical (i.e., blistering in terms of vesicular and bullous appearance), eczematous, prurigo-type, and other presentations including urticarial (prodromal) phenotypes (Figure 1). For the latter three mentioned groups, we conditioned absence of vesicles or bullae as reported by the patient and documented in the patient’s medical records.

### 3.2. Dermatopathological Characteristics

**Figure 2:**
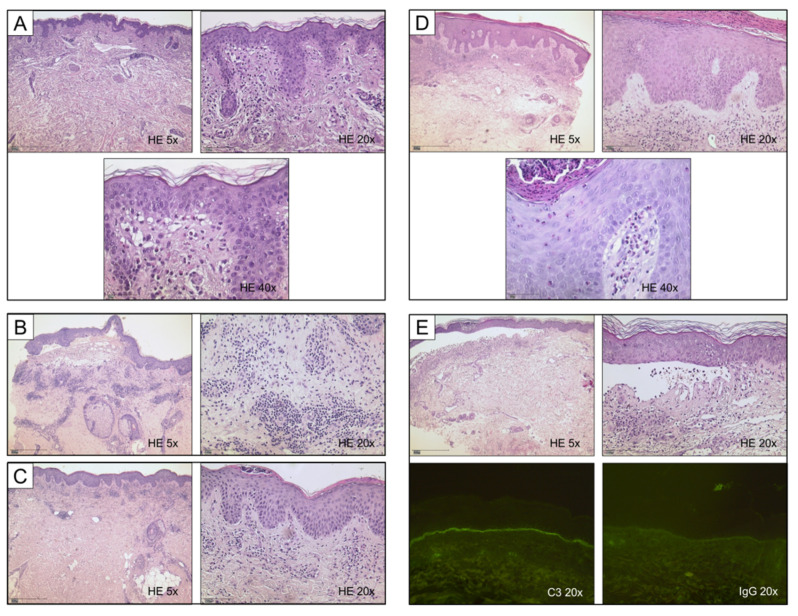
Histological synopsis of different manifestations of bullous pemphigoid; cases correspond to Figure 1. (**A**) Unremarkable epithelium, yet abundant eosinophils and marked papillary edema. (**B**) Fully developed subepidermal blister containing numerous eosinophils and erythrocytes; note a remarkably dense perivascular lymphocytic infiltrate. (**C**) Superficial perivascular dermatitis with psoriasiform hyperplasia and marked parakeratosis; subcorneal neutrophils are testimony of previous scratching which resulted in ulceration. (**D**) Histologic features of both psoriasis (acanthotic epidermis with hypogranulosis and compact parakeratosis) and marked tissue eosinophilia, note eosinophilic spongiosis. (**E**) Fully developed subepidermal blister with predominance of neutrophils; note intracorneal pustule in the detached epidermis. Immunofluorescence confirmed diagnosis of BP in the respective patients. Linear deposits of C3 and immunoglobulins are the prerequisite diagnostic criterion for BP, especially with unusual clinical appearance.

**Figure 3:**
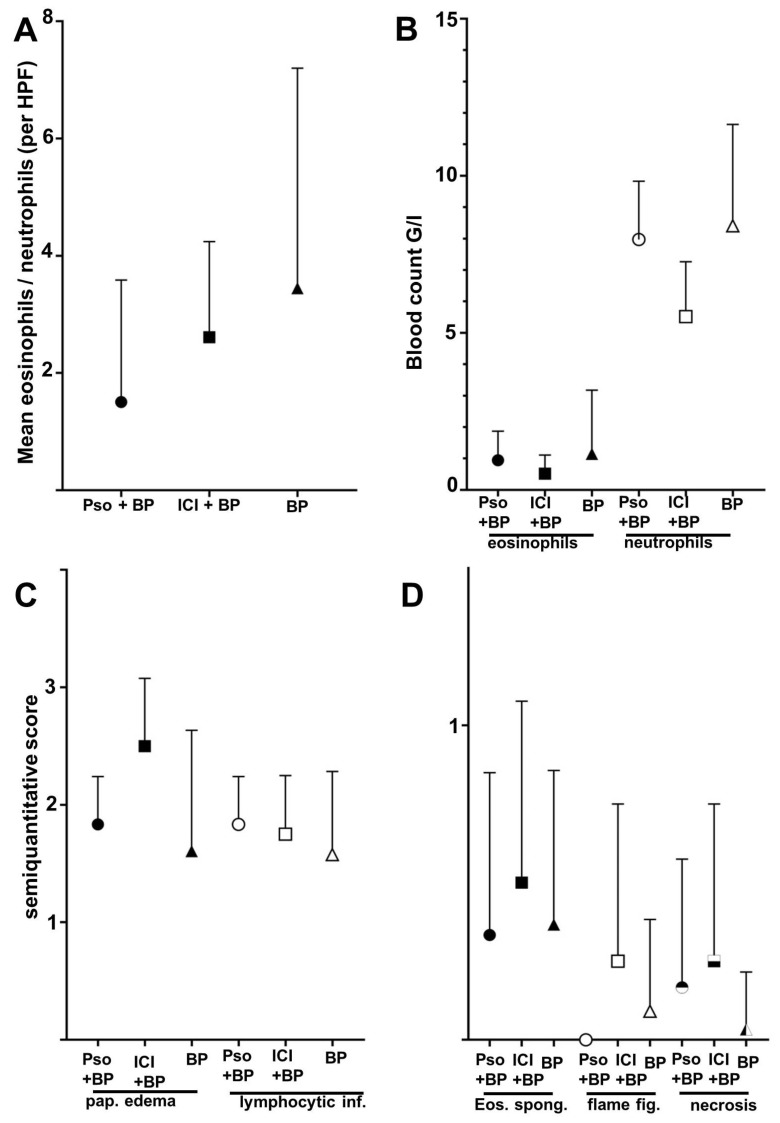
Differences of histolopathologic and laboratory features between the groups of BP in psoriatic patients (Pso + BP), BP in patients receiving immune checkpoint inhibitors (ICI + BP), and the control group of idiopathic BP (BP). Whiskers indicate standard deviation and are shown one-sided only to avoid negative values (**A**). Tissue ratio of eosinophils/neutrophils as assessed as mean of four random high-power fields (HPF) per section. (**B**) Total blood count of eosinophils and neutrophils of the patients at the time of biopsy. (**C**) Semi-quantitative score of papillary edema (pap. edema) and lymphocytic infiltrates (lymphocytic. inf.) (0 = absent, 1 = low, 2 = moderate, 3 = strong). (**D**) Frequency of eosinophilic spongiosis (Eos. spong.), flame figures (flame fig.) and necrosis (0 = absent, 1 = present). None of the shown comparisons reached statistical significance (Mann–Whitney test).

The analyzed specimens showed considerable histopathological variability (Figure 2A–E). As expected, the majority of idiopathic BP showed a higher density of eosinophils than neutrophils in the upper dermis and had a ratio of eosinophils to neutrophils (E-N ratio) greater than one (Figure 3A). Still, eleven biopsies showed a higher density of neutrophils. In addition, 57.1% of skin biopsies from psoriatic patients had an E-N ratio smaller than one, which included both typical and eczematous clinical phenotypes. Deviating from our hypothesis, all specimens of BP in patients receiving ICB showed an E-N ratio greater than one.

The group of idiopathic BP showed both the highest mean number of peripheral eosinophils (1.14 G/L) when compared to psoriatic patients (0.94) and ICB patients (0.54), and also peripheral neutrophils (8.41 G/L, 7.97 G/L and 5.52 G/L, respectively) (Figure 3B). We further evaluated histopathological characteristics of the three groups of BP. Papillary edema as a sign of acuity was strongest in the group of ICB-associated BP (mean score 2.50), while idiopathic BP lesions reached a mean score of 1.61. There were no relevant differences in the density of the perivascular lymphocytic infiltrate (scores ranging from 1.58–1.83 among the groups). Eosinophilic spongiosis, defined as even a singular eosinophil present in the epidermis with spongiotic distension of keratinocytes, was found in 33–50% of cases in the three groups; the finding was most common within the ICB group. The same was found for flame figures; still, based on the small size of our groups, these findings were not statistically significant. Epidermal necrosis was rarely detected with one case in each group, which rendered a lower relative number in the idiopathic BP group.

### 3.3. Literature Review

To compare our results with previously published reports, we performed a thorough literature review including different commonly used PD1 and PDL1 inhibitors. We identified 71 reported cases (Table 2) with a male predominance (70.4%) and a mean age of 70.2 years (SD 11.2 years). The most often mentioned causative agents were pembrolizumab in 35 cases and nivolumab in 29 cases. Other PD1/PDL1 inhibitors were less frequently identified as culprit drugs (cemiplimab: 1; atezolizumab: 4; durvalumab: 2). CTLA4 inhibitors alone or in combination with PD1 inhibitors were mentioned in further cases (ipilimumab: 13; tremelimumab: 1). The largest number of case reports included patients with metastatic melanoma (31) or non-small cell lung cancer (NSCLC) (24). Other malignancies were renal cell carcinoma (RCC) (5), cutaneous squamous cell carcinoma (cSCC) (2), head and neck squamous cell carcinoma (HNSCC) (2), and singular cases of other diagnoses (7).

From a clinical perspective, blistering was reported in 67/71 cases (94.3%); however, in many cases, urticarial or eczematous skin lesions preceded development of blisters or bullae. Mucosal involvement of any type was reported in 18 cases (25.3%), whereas 31 case reports did not include information in this regard and another 20 case reports stated that there was no mucosal involvement. Unfortunately, information regarding comorbidities, and comedications was incomplete. In general, there was a striking predominance of Caucasian and Asian patients.

We analyzed the literal descriptions of the histology reports to deduct that eosinophils were predominant in 56 cases (78.8%) compared to only six cases (8.4%) with neutrophils as predominant inflammatory cells. From a histopathological perspective, one can assume that most biopsied lesions were fully developed blisters, as 53 (74.6%) showed clefting. Moreover, perivascular lymphocytic infiltrates were described in 26 (36.6%) cases. Eosinophilic spongiosis was a rare feature in eight cases and eosinophilic tagging along the dermal –epidermal junction was described in three cases. Flame figures were not mentioned at all, and epidermal necrosis was present in only two cases. All but nine case reports included information regarding DIF. The vast majority was IgG positive (49/62; 79%); only three cases displayed IgA (4.8%), and nine cases displayed C3 deposits only (14.5%).

## 4. Discussion

The most striking findings of this study were that ICB-associated BP was more likely to occur in male patients and that patients were significantly younger. This type of gender-gap appears counter-intuitive at first, as female patients show a higher incidence of most autoimmune diseases and most commonly show a higher incidence of immune-related adverse events to immune-stimulatory drugs, such as vaccines or ICB [86]. One could assume that specific immunological effects of the interaction between the immune-stimulatory drugs and cancer cells result from altered levels of sex hormones. Other intrinsic or extrinsic factors might also be accountable. This topic should be investigated further, ideally as part of clinical trials. When taking a closer look at clinical subtypes of BP, peculiar differences between the groups were noted. Mucosal involvement tended to occur more often in ICB-associated BP. One explanation might be that a broad activation of adaptive immune responses could potentially render the way to epitope spreading once an anti-epithelial response is established. In the analyzed cases of our center, all analyzed ICB-associated BP showed a “typical” clinical appearance with blistering, whereas one third of the psoriatic and 12% of the control group showed eczematous lesions only. Admittedly, clinical variation might also result from timing of consultation and biopsy after perception of initial symptoms. This time was estimated according to the patient records to be 5.5 months in the psoriatic group versus 3.5 months in the ICB-associated BP group and 3.75 months in the control group, respectively. As a consequence of the variable clinical aspects, otherwise inexplicable pruritus should trigger a diagnostic workup including conventional histology and DIF, especially in elderly patients.

The patients reported in the literature had often suffered from preceding urticarial or eczematous lesions for weeks to months before finally developing vesicles or bullae. This finding deserves attention as it might point towards a commonly delayed diagnostic workup. As not all patients necessarily reach the bullous phase, underdiagnosis of ICB-associated-BP might be common. Publication bias must be considered as well, as potentially more severe cases could have been selected for publication. Another important aspect of this analysis is that ICB-associated BP seems to occur with various solid cancers and is not limited to melanoma. Clearly, there is a need for prospective trials to gain a better understanding of the exact frequency of BP associated with ICB.

Our initial hypothesis was invalidated as no statistically significant differences could be found regarding histopathological findings between the groups of BP with or without psoriasis and association with ICB, which might be due to small sample size. Many of the published case reports did not include sufficient information regarding histologic findings. Yet, the vast majority mentioned eosinophils as the predominant inflammatory cell type. In one case report, the histologic description of ICB-induced BP in a psoriatic patient was even eosinophilic only [48], which is in stark contrast with the report of BP in psoriatic patients not undergoing ICB, provided by Inamura et al. [20]. Another lead of interest is that eight of the case reports mentioned vacuolization as part of interface-dermatitis. Some authors speculate that a lichenoid type of inflammation, which is a classical finding in ICB-associated dermatitis as result of a Th1-polarized inflammation, might trigger unmasking of antigens of the basal layer, which would predispose to development of BP [81]. For that reason, we also included cases of lichen planus pemphigoides in this analysis. After all, ICB-induced BP might represent a continuum with ICB-induced lichenoid tissue reactions. As stated above, BP might be underdiagnosed in this patient population, as DIF is not routinely performed when vesicles or bullae are absent.

The methodology of this study is purely retrospective, which limits its validity. The most obvious potential bias concerning our analysis of the inflammatory infiltrate is the age of the biopsied lesions. Higher numbers of neutrophils might simply reflect older lesions rather than a different pathogenetic mechanism. Both early and fully developed lesions were included in this study altogether and pretreated lesions were not excluded, which represents a potential confounder. However, the treating physicians in our center are trained in performing biopsies of the freshest lesions available, and therefore a systematic overestimation towards one over the other groups is unlikely. Even though all cases were confirmed by DIF, it is noteworthy that data regarding the identified antigen was not available in all cases. We cannot rule out completely that singular cases of anti-laminin-gamma-1 pemphigoid or deep lamina lucida (anti-p105) pemphigoid were included in this study, which may display greater abundance of neutrophils.

It is important to compare our results with previous works. A retrospective analysis of twelve cases was not included due to lack of clinical and histopathological information [87]. Another retrospective analysis of twelve cases from six German centers was not included in Table 2 either [88]. These authors deducted that ICB-induced BP might have distinct similarities to gliptin-induced BP [89,90], and tended to be milder than classical BP as most cases could readily be controlled with topical corticosteroids. Similar to our results, the majority of patients (83.3%) were male and mucosal involvement was common (16.6%). The age ranged from 62–80, with a median of 76 years. Histological descriptions were only available for seven patients and the majority showed eosinophils. Interestingly, in both articles, the authors speculated that ICB-induced BP might be a favorable prognostic factor in melanoma patients, which would be comparable to other cutaneous irAE (e.g., vitiligo). Another retrospective single-center analysis included seven cases of ICB-induced BP and was not included in Table 2 either, but it showed similar findings to our study [91]. The histological findings resembled idiopathic BP with subepidermal clefting and preponderance of eosinophils. Through DIF, IgG was the most often identified immunoglobulin subtype. The male to female ratio was 4:3, and one patient out of seven displayed mucosal involvement (14.2%). Interestingly, the authors estimated the incidence of ICB-associated BP to be as high as 1%, and the patients were treated for a variety of tumors, with melanoma and lung cancer being the most frequent.

A retrospective multicenter cross-sectional study aimed to define differences in diagnostics and therapeutic interventions between ICB-associated BP and idiopathic BP [92]. In this study, 15 ICB-associated BP were identified, with only 20% of the patients being female. Interestingly, subepidermal clefting on histopathology was less likely than in idiopathic BP. The authors identified persisting pruritus in the absence of skin symptoms as a hallmark finding in ICB-BP, which corresponds to the equivalent of a longer prodromal phase. It was already concluded by the same group that early management is beneficial and that misdiagnosis may lead to prolonged immunosuppression and consecutive morbidity [93].

## 5. Conclusions

In summary, our study confirmed peculiar clinical differences between ICB-associated BP and idiopathic BP; specifically, a larger proportion of male patients and a more frequent involvement of oral mucosa. Our data was insufficient to detect significant differences regarding histopathologic findings of these conditions; we also could not confirm differences in histopathologic findings of BP patients with psoriatic background. Both the exact molecular mechanisms of ICB-associated BP and the best suitable treatment options remain to be specified in detail.

## Figures and Tables

**Table 1:** Patient characteristics. Patients with BP receiving ICB were younger and had a higher daily intake of prescribed drugs; diabetes mellitus type II was less common.

Diagnosis	Age Mean (SD), Range	Clinical Appearance	Mucosal Involvement	DIF	DM Type II	Active Neoplastic Disease	Drugs Mean (SD) Range	DPP4 Inhibitor	Treatment
BP and psoriasis (*n* = 6; F = 3/M = 3)	81.2 (±8.8) 66–89	Typical: 4 Eczematous: 2	0/6	IgM: 0 IgG: 5 IgA: 0 C3 only: 1	2/6	1/6	6.0 (±1.8) 4–8	0/2	SC: 2 Doxy: 3 MMF: 0 MTX 1
BP and ICB (*n* = 4; F = 1/M = 3)	73.3 (±5.7) 68–80	Typical: 4	2/4	IgM: 0 IgG: 4 IgA: 0 C3 only: 0	0/4	4/4	8.3 (±4.2) 4–14	0/0	SC: 2 Doxy: 1 MMF: 0 Dapsone 1
BP (*n* = 33; F = 20/M = 13)	81.1 (±11.0) 58–97	Prodromal: 1 Typical: 27 Eczematous: 4 Prurigo type: 1	2/33	IgM: 3 IgG: 31 IgA: 2 C3 only: 0	12/33	1/33	7.0 (±3.1) 1–13	4/12	SC: 22 Doxy: 20 MMF: 8 Dapsone: 3 Dupilumab: 1

Abbreviations: BP, bullous pemphigoid; ICB, immune checkpoint blockade; SD, standard deviation; DIF, direct immunofluorescence; DM, diabetes mellitus; DPP4, dipeptidyl peptidase-4 inhibitor; SC, systemic corticosteroids; doxy, doxycycline; MMF, mycophenolate mofetil; MTX, methotrexate.

**Table 2:** Overview of case reports of BP and lichen planus pemphigoides (LPP) in patients receiving ICB with a focus on clinical and histopathological findings (alphabetical order of first authors, as of May 2021). Cases involving both programmed cell death protein 1/ligand 1 inhibitors (PD1/PDL1) and cytotoxic T-lymphocyte associated protein 4 (CTLA4) inhibitors were included. The manuscripts were manually scanned for relevant comorbidity and specific information with regard to BP (e.g., diabetes type II); tumor-related symptoms are not listed (e.g., renal failure in association with urothelial cancer), whereas concomitant side effects of ICB are listed.

Case #	Sex	Age	ICI	Indication	Clinical Appearance	Mucosal Involvement	DIF	Specific Histologic Findings	Comorbidity/Specifics	Reference
1	M	80	Pembro	NSCLC	Typical; other: cellulitis-like appearance	Erosions on lips and oral mucosa	IgG	Subepidermal blister inflammatory cells	None reported	Adachi et al. [29]
2	M	48	Nivo	Melanoma	Typical	None	NA	Subepidermal blister Eosinophils and lymphocytes in upper dermis	Polymorphous adenoma of the parotid, hyperlipidemia, smoking	Anastasopoulou et al. [30]
3	F	75	Nivo	Melanoma	Typical	None	IgG	Eosinophils and lymphocytes in infiltrate	None reported	Aoki et al. [31]
4	M	73	Pembro	Melanoma	Typical; other: hyperkeratotic crateriform lesions	None	IgG	Subepidermal cleft, tagging of eosinophils along dermal–epidermal junction	None reported	Bandino et al. [32]
5	M	90	Pembro, then Nivo	Melanoma	Other: localized blistering and ulcer	None	NA	Subepidermal vacuolization with eosinophilic spongiosis, increased dermal eosinophils	None reported	Bandino et al. [32]
6	M	72	Ipi, then Pembro	Melanoma	Typical	Severe involvement	NA	Subepidermal cleft, eosinophils, perivascular mixed infiltrate	Preexisting BP flare with ICB (Ipi > Pembro)	Beck et al. [33]
7	M	73	Pembro	NSCLC	Typical	Oral mucosa and throat	IgG	Early: spongiosis, irregular acanthosis, focal exocytosis of lymphocytes and eosinophils, mild superficial perivascular inflammation with eosinophilis Late: subepidermal blisters with eosinophils	Former smoking, zoster, irAE: pneumonitis and adrenal insufficiency	Cardona et al. [34]
8	M	75	Ipi, then Pembro	Melanoma	Typical	None	IgG	Similar to Cardona et al.	None reported	Carlos et al. [35]
9	F	77	Pembro	NSCLC	Typical	Gingival mucosa	IgG	Subepidermal cleft with fibrin and eosinophils, eosinophils and lymphocytes in dermal papillae	Pancreatitis	Cosimati et al. [36]
10	M	74	Nivo	NSCLC	Typical	Oral mucosa	C3 only	Subepidermal cleft with eosinophils and fibrin, neutrophils in the upper dermis	None reported	Cuenca-Barrales et al. [37]
11	F	77	Nivo	NSCLC	Typical	None	IgG	Eosiniophilic spongiosis, mixed dermal infiltrate with eosinophilia	Inverse psoriasis, diabetes mellitus, hypertension, COPD, depression	Damsky et al. [38]
12	F	65	Durva and Tremi	NSCLC	Typical	None	IgG	Subepidermal cleft, epidermal necrosis, perivascular infiltrate of lymphocytes and eosinophils	None reported	Fontecilla et al. [39]
13	M	64	Pembro	Urothelial carcinoma	Typical	None	IgG	Subepidermal cleft with eosinophils, perivascular eosinophils and lymphocytes	Preexisting BP flare with Pembro	Garje et al. [40]
14	M	63	Nivo	NSCLC	Typical	None	IgG, IgA	Subepidermal blistering with infiltrating lymphocytes and eosinophils	COPD	Grän et al. [41]
5	M	78	Nivo	RCC	Typical	Oral and genital mucosa	C3 only	Separation of epidermis from dermis at the basement membrane	Onset of symptoms associated with radiotherapy	Grimaux et al. [42]
16	M	72	Pembro, then Ipi	Melanoma	Typical	None	IgG	Subepidermal blister, perivascular lymphocytic infiltrates, multiple eosinophils	irAE: grade 4 diarrhoea	Hanley et al. [43]
17	M	60	Pembro	NSCLC	Typical	None reported	IgG	NA	None reported	Hara et al. [44]
18	M	70	Ipi, then Pembro, then Nivo	Melanoma	Typical	None	IgG	Subepidermal cleft, numerous eosinophils	Blistering localized and associated with radiotherapy irAE: hypophysitis	Hirotsu et al. [45]
19	F	56	Pembro	Endometrial carcinoma	Prurigo-type	None	IgG	Subepidermal cleft, eosinophils	irAE: sarcoidal granulomatous panniculitis preceding BP	Honigman et al. [46]
20	M	68	Pembro	Melanoma	Typical/ Prurigo-type	Single erosion of oral mucosa	IgG	Early: aspects of grover’s disease Late: mild papillary dermal chronic inflammation with scattered eosinophils	Non-melanoma skin cancer, irAE: vitiligo	Hwang et al. [47]
21	M	72	Pembro, then Ipi	Melanoma	Typical	None	IgG	Subepidermal cleft with eosinophils, neutrophils and fibrin	Non-melanoma skin cancer, irAE of Ipi: pneumonitis	Hwang et al. [47]
22	M	63	Nivo	HNSCC	Typical	Mucosal blistering	IgG	Subepidermal blister, mixed inflammatory infiltrate of neutrophils and eosinophils	None reported	Jour et al. [48]
23	M	68	Pembro	Melanoma	Typical	None	IgG	Perivascular inflammation and eosinophils in the blister cavity	Psoriasis vulgaris, worsening with Pembro	Jour et al. [48]
24	F	74	Nivo + Ipi	Urothelial carcinoma	Typical	None reported	IgG	Subepidermal blister with eosinophils	None reported	Jour et al. [48]
25	F	73	Nivo	NSCLC	Typical	None	NA	Subepidermal blister with eosinophils, dermal lymphocytic infiltration	None reported	Jour et al. [48]
26	M	67	Pembro	NSCLC	Typical	Gingival erosions	IgG	Spongiosis, lymphocytic exocytosis, perivascular lymphocytic infiltrate, numerous eosinophils	Preexisting BP in remission-flare with Pembro	Kaul et al. [49]
27	M	70	Nivo	RCC	Typical	Oral mucosa	C3 only	sub- and intra-epidermal blister with eosinophils, eosinophils in the dermis	Blistering limited to sun-exposed areas	Kluger et al. [50]
28	M	87	Atezo	Urothelial carcinoma	Typical	None	IgG	Subepidermal blister, paucicellular infiltrate	None reported	Kosche et al. [51]
29	M	35	Nivo, then Ipi	Melanoma	Typical	None reported	IgG	Subepidermal blister, moderate eosinophilic infiltration of the upper dermis	None reported	Kuwatsuka et al. [52]
30	M	60	Nivo	RCC	Typical	None reported	IgG	Subepidermal cleft, perivascular and interstitial mixed cell infiltrate, lymphocytes and eosinophils	None reported	Kwon et al. [53]
31	F	65	Pembro	Merkel-cell carcinoma	Other: lichenified papules and plaques	None reported	IgG	Lichenoid and vacuolar epidermal interface alteration with associated dyskeratotic keratinocytes and eosinonophils	Diagnosis of LPP favored over BP	Kwon et al. [54]
32	M	82	Atezo	cSCC	Typical	None	IgG	Pauci-inflammatory subepidermal blister	Blistering in sun-exposed areas	Leavitt et al. [55]
33	M	30–39	Nivo	HNSCC	Typical	Ulcers on oral mucosa	IgG	Subepidermal blister with a mixed inflammatory infiltrate, many eosinophils	None reported	Lee et al. [56]
34	F	82	Ipi, then Pembro	Melanoma	Typical	None reported	NA	Subepidermal blister, superficial perivascular and interstitial inflammatory infiltrate of lymphocytes, eosinophils and occasional neutrophils	None reported	Lomax et al. [57]
35	F	72	Nivo	NSCLC	Typical	None	IgG	Perivascular lymphocytic and eosinophilic infiltrate	Laryngeal cancer, successfully treated with chemoradiation	Lopez et al. [58]
36	F	80	Nivo	NSCLC	Typical	None reported	C3 only	Vacuolar changes at the dermal–epidermal junction with eosinophilic infiltration in the dermis	None reported	Maya et al. [59]
37	M	63	Pembro	Melanoma	Typical	None reported	C3 only	Subepidermal blister, superficial dermal inflammatory infiltrate with lymphocytes and eosinophils, intraepithelial eosinophils	None reported	Mochel et al. [60]
38	M	62	Nivo	RCC	Typical	None reported	IgG	Subepidermal blister, dermal lymphocytic infiltrate with numerous eosinophils	None	Munera-Campos et al. [61]
39	M	84	Pembro	NSCLC	Typical	None reported	IgG	Subepidermal blister with moderate eosinophil and neutrophil infiltration of the upper dermis, eosinophilic spongiosis	None reported	Muto et al. [62]
40	M	80	Ipi, then Nivo	Melanoma	Typical	Initially none, later erosions and vesicles on buccal mucosa	IgG	Ulcerated and inflamed subepidermal vesicular dermatitis with eosinophils	None	Naidoo et al. [63]
41	F	78	Ipi, then Durva	Melanoma	Typical	Buccal mucosa	IgG	Subepidermal cleft	None	Naidoo et al. [63]
42	M	85	Nivo	NSCLC	Typical	None reported	IgG	Subepidermal bullous dermatitis with eosinophils	None	Naidoo et al. [63]
43	M	79	Pembro	Cholangio-carcinoma	None reported	None reported	NA	Marked infiltration of CD4+, CD8+ and CD163+ cells	None reported	Nakai et al. [64]
44	F	75	Nivo	Melanoma	Typical	Faint striae on cheeks, oral paresthesia	IgG	Subepidermal fissuring with a dense inflammatory infiltrate and colloid bodies, necrotic epithelium with a dense perivascular and periadnexal lymphocytic infiltrate	Hypertension, hypothyreodism	Niebel et al. [65]
45	M	62	Nivo	RCC	Typical	None	IgG	Subepidermal cleft with eosinophils, eosinophils tagging the intact dermal–epidermal junction	Hypertension, coronary artery disease, chronic kidney disease, hereditary focal segmental glomerulosclerosis	Palla et al. [66]
46	M	42	Ipi, then Pembro	Melanoma	Typical	None	C3 only	Eosinophil-predominant inflammatory cell infiltration, particularly in the interstitium and perivascular space	None	Parakh et al. [67]
47	F	79	Pembro	NSCLC	Typical	None reported	IgG	Lichenoid dermatitis with subepidermal blister formation	None reported	Qiu et al. [68]
48	M	67	Nivo	Melanoma	Typical	None reported	IgG	Subepidermal bullous dermatosis	None reported	Ridpath et al. [69]
49	F	56	Ipi, then Pembro	Melanoma	Typical	None reported	IgG	Subepidermal blister with mononuclear cells and eosinophils in the papillary dermis	Hypothyreodism, irAE: primary adrenal insufficiency	Rofe et al. [70]
50	M	58	Atezo	NSCLC	Typical	None reported	IgG	Subepidermal blister with eosinophils	None reported	Russo et al. [71]
51	F	69	Nivo	Melanoma	Typical	None reported	IgG	Moderate lymphohistiocytic dermal infiltrate	irAE: thyreoiditis	Sadik et al. [72]
52	M	57	Nivo	NSCLC	Typical	None reported	IgG	Vacuolar degeneration with apoptotic keratinocytes and prominent eosinophil infiltration at the epidermal junction and band-like infiltration of lymphocytes	Diagnosis of LPP favored over BP; hand-foot syndrome with chemotherapy, diabetes treated with vildagliptin	Sato et al. [73]
53	M	64	Pembro	Melanoma	Typical	Oral mucosa	C3 only	Subepidermal blistering, few eosinophils and lymphocytic infiltrate	Diagnosis of LPP favored over BP	Schmidgen et al. [74]
54	F	72	Pembro	Melanoma	Eczematous, singular vesicle	None reported	IgA and IgG	Subepidermal split, dense eosinophilic infiltrate in the dermis	None reported	Schwartzman et al. [75]
55	M	82	Ipi and Nivo	Melanoma	Typical	None reported	IgA and IgG	Subepidermal split with predominantly eosinophils and scattered neutrophils	None reported	Schwartzman et al. [75]
56	M	68	Nivo	NSCLC	Typical	None reported	“consistent with BP”	Psoriasiform dermatitis	irAE: thyroiditis, dermatitis, and nephritis	Schwartzman et al. [75]
57	F	76	Atezo	NSCLC	Blistering + other: violaceous, flat-topped polygonal papules and plaques	White reticular lesions of the oral mucosa	C3 only	Hypergranulosis, subepidermal blister, mixed infiltrate of eosinophils and lymphocytes, vacuolar degeneration at the dermoepidermal junction, band-like lymphocytic infiltration in the upper dermis	Diagnosis of LPP favored over BP; gallbladder cancer, hypercholesterinemia	Senoo et al. [76]
58	M	76	Pembro	NSCLC	Typical	None reported	NA	Subepidermal vesicles with underlying mixed-cell infiltrates including numerous eosinophils	None reported	Sharma et al. [77]
59	M	78	Nivo	Melanoma	Eczematous	Desquamative gingivitis	IgG	Eosinophilic Spongiosis, eosinophil tagging of the dermal–epidermal junction	None reported	Singer et al. [78]
60	M	78	Pembro	Esophageal carcinoma	Eczematous	None	C3 only	Mixed spongiotic, micropustular, and interface dermatitis with numerous eosinophils	None reported	Singer et al. [78]
61	M	62	Pembro	NSCLC	Eczematous	None reported	IgG	Subacute spongiosis and papillary dermal chronic inflammation with numerous eosinophils	None reported	Singer et al. [78]
62	M	58	Pembro	Melanoma	Eczematous	None reported	NA	Acute and chronic inflammation suggestive of component of hypersensitivity reaction	None reported	Singer et al. [78]
63	M	80–89	Nivo	NSCLC	Typical	Gingival bulla	IgG	Subepidermal vesicle with numerous eosinophils	Delirium, osteopenic compression fractures	Sowerby et al. [79]
64	F	87	Nivo	NSCLC	Typical	None reported	(-)	Subepidermal bullous lichenoid eruption with eosinophils	Diagnosis of LPP favored over BP; congestive heart failure, coronary artery disease, chronic kidney disease, hypertension	Strickley et al. [80]
65	F	72	Pembro	NSCLC	Typical	None reported	IgG	Early: compact orthokeratosis and hypergranulosis, vacuolar alteration of the basal layer and dermal lymphocyte infiltration Late: subepidermal blister with eosinophilic infiltration	Diabetes treated with sitagliptin and teneligliptin	Sugawara et al. [81]
66	F	86	Pembro	Melanoma	Typical	None reported	NA	Subepidermal bulla with eosinophils	None reported	Sun et al. [82]
67	M	82	Pembro	Melanoma	Typical	None reported	IgG	Subepidermal bulla and inflammatory infiltrate with eosinophils	Chronic lymphocytic leukemia, renal cell carcinoma, diabetes	Sun et al. [82]
68	M	64	Pembro	Melanoma	Typical	None reported	IgG	Superficial perivascular and interstitial inflammation dominated by eosinophils, beginning dermoepidermal bulla	Urolithiasis	Thomsen al. [83]
69	M	71	Ipi, then Pembro	Melanoma	Typical	None reported	(-)	Dermoepidermal bulla	Pneumonia, myocardial infarction	Thomsen et al. [83]
70	M	68	Cemi	cSCC	Typical	None	IgG	Subepidermal blister, eosinophilic spongiosis, dermal eosinophilia	Non-melanoma skin cancer	Virgen et al. [84]
71	M	65	Pembro	Melanoma	Typical	None reported	IgG	Subepidermal blister with eosinophils	irAE: vitiligo	Wada et al. [85]

Abbreviations: DIF, direct immunofluorescence; LPP, lichen planus pemphigoides; NSCLC, non-small cell lung cancer; RCC, renal cell carcinoma; HNSCC, head and Neck squamous cell carcinoma; cSCC, cutaneous squamous cell carcinoma; NA, not available; irAE, immune-related adverse events; Pembro, pembrolizumab (PD1); Nivo, nivolumab (PD1); Cemi, cemiplimab (PD1); Treme, tremelimumab (CTLA4); Ate, atezolizumab (PDL1); Ipi, ipilimumab (CTLA4); Durva, durvalumab (PDL1).

## Data Availability

Further data may be obtained from the corresponding author upon reasonable request.
